# Kosteneffizienz von Impfungen: Über die Komplexität gesundheitsökonomischer Analysen zur Influenza‑, SARS-CoV-2- und RSV-Impfung

**DOI:** 10.1007/s00103-025-04022-8

**Published:** 2025-03-04

**Authors:** Peter Klimek

**Affiliations:** 1 Center for Medical Data Science, Institute of the Science of Complex Systems, Medizinische Universität Wien, Spitalgasse 23, 1090 Wien, Österreich; 2 Complexity Science Hub, Wien, Österreich; 3 Supply Chain Intelligence Institute Austria (ASCII), Wien, Österreich; 4 Department of Clinical Neuroscience, Division of Insurance Medicine, Karolinska Institutet, Stockholm, Schweden

**Keywords:** Kosteneffektivität, Impfungen, Influenza, SARS-CoV‑2, Respiratorisches Synzytial-Virus (RSV), Cost effectiveness, Vaccinations, Influenza, SARS-CoV‑2, Respiratory syncytial virus (RSV)

## Abstract

Die Frage nach der Kosteneffektivität medizinischer Interventionen ist eine der zentralen Fragen der Gesundheitsökonomie. Dieses narrative Review untersucht die Kosteneffektivität von Impfungen gegen Influenza, SARS-CoV‑2 und das respiratorische Synzytial-Virus (RSV) unter Berücksichtigung aktueller gesundheitsökonomischer Analysen. Die jährliche Influenza-Impfung und die Auffrischungsimpfung gegen SARS-CoV‑2 in den Jahren 2023 und 2024 erweisen sich, insbesondere in Hochrisikogruppen, als kosteneffektiv und teilweise sogar kostensparend. Für die RSV-Impfung, die 2023 zugelassen wurde, ist die Kosteneffektivität weniger klar. Sie hängt stark von der Altersgruppe und der Bereitschaft ab, für ein gewonnenes qualitätsadjustiertes Lebensjahr (QALY) zu zahlen. Die Analyse zeigt, dass die Bewertung von Impfungen eine erhebliche Datenmenge erfordert. Modellrechnungen zu Impfungen müssen neben direkten Schutzwirkungen auch indirekte Effekte, wie die Reduzierung von Übertragungen in der Bevölkerung bei höheren Impfraten, berücksichtigen. Sensitivitätsanalysen verdeutlichen, dass Faktoren wie Impfstoffkosten, Effektivität und Krankheitsinzidenz entscheidenden Einfluss auf die Kosteneffektivität haben können. Eine der größten Herausforderungen in gesundheitsökonomischen Analysen ist die Fragmentierung von Gesundheitsdaten in vielen Ländern, was umfassende und präzise Bewertungen erschwert. Initiativen wie der europäische Gesundheitsdatenraum könnten hier Abhilfe schaffen und eine evidenzbasierte Entscheidungsfindung in der Gesundheitspolitik unterstützen. Insgesamt bleibt die Kosteneffektivität von Impfungen abhängig von zahlreichen Faktoren, wobei insbesondere die SARS-CoV-2- und Influenza-Impfungen in den betrachteten Szenarien eine positive Bewertung erhalten.

## Abstract (en)

The question of the cost effectiveness of medical interventions is one of the central issues in health economics. This narrative review examines the cost effectiveness of vaccination against influenza, SARS-CoV‑2 and respiratory syncytial virus (RSV) considering current health economic analyses. The annual influenza vaccination and the booster vaccination against SARS-CoV‑2 in 2023 and 2024 are proving to be cost effective and in some cases even cost saving, especially in high-risk groups. The cost effectiveness of the RSV vaccination, which was approved in 2023, is less clear. It strongly depends on the age group and the willingness to pay for a quality-adjusted life year (QALY) gained. The analysis shows that the evaluation of vaccinations requires a considerable amount of data. In addition to direct protective effects, model calculations on vaccinations must also consider indirect effects, such as the reduction of transmission in the population with higher vaccination rates. Sensitivity analyses make it clear that factors such as vaccine costs, effectiveness and disease incidence can have a decisive influence on cost effectiveness. One of the biggest challenges in health economic analyses is the fragmentation of health data in many countries, which makes comprehensive and precise assessments difficult. Initiatives such as the European Health Data Space could help and support evidence-based decision making in health policy. Overall, the cost effectiveness of vaccinations remains dependent on numerous factors, with SARS-CoV‑2 and influenza vaccinations receiving a positive assessment in the scenarios analysed.

## Hintergrund

Ob sich medizinische Interventionen in einer bestimmten Bevölkerungsgruppe lohnen, ist eine der zentralen Fragestellungen in der Gesundheitsökonomie und insbesondere von Kosteneffektivitätsanalysen [1, 2]. Für solche Analysen werden 2 oder mehr Behandlungsoptionen verglichen, z. B. der aktuelle Versorgungsstandard mit einer neuen Therapieoption oder, im Falle von Impfempfehlungen, der Vergleich zwischen einer Nichtimpfung und einer Impfung.

Diese Untersuchungen erfordern eine Bewertung des Verhältnisses zwischen den Kosten- und Nutzenunterschieden der Strategien. Die Kosten umfassen sowohl direkte (z. B. Therapiekosten) als auch indirekte Kosten (z. B. Kosten durch Folgeerkrankungen bei Unterlassung der Therapie). Der Nutzen wird meist in qualitätsadjustierten Lebensjahren (QALYs) gemessen [3]. QALYs bewerten sowohl die Anzahl der gewonnenen Lebensjahre als auch deren Qualität.

Ein zentraler Wert zur Bewertung dieses Verhältnisses ist das inkrementelle Kosten-Nutzen-Verhältnis („incremental cost-effectiveness ratio“, ICER), definiert als das Verhältnis der durchschnittlichen Kostenunterschiede in der relevanten Population zu den Unterschieden in den Outcomes. In der Praxis führt dies oft zur Frage: Wie viel Euro ist man bereit, für ein QALY auszugeben [4]?

Die wenigsten Länder legen klare Schwellenwerte fest. Eine Ausnahme bildet das Vereinigte Königreich, wo eine lange Tradition von Kosteneffektivitätsanalysen besteht. Der Schwellenwert für die Kosteneffektivität liegt dort bei etwa 30.000 Pfund (ca. 50.000 €) pro QALY [5]. Therapien, die diesen Wert überschreiten, gelten als nicht kosteneffektiv. In anderen reichen Ländern wird manchmal auch ein inoffizieller Schwellenwert im Bereich von 100.000 US-Dollar genannt [6].

Die Weltgesundheitsorganisation (WHO) schlägt in ihrer CHOICE-Initiative (CHOosing Interventions that are Cost-Effective) einen länderspezifischen Grenzwert vor, der in einem Bereich vom 1‑ bis 3‑Fachen des Bruttoinlandprodukts pro Kopf (BIP p.K.) liegt [7]. Es bestehen jedoch Bedenken, dass diese populäre, weil einfache Regel insbesondere in Ländern mit niedrigem oder mittlerem Einkommen in zu hohen Schwellwerten mündet, nicht zuletzt da sie keine gesundheitlichen Opportunitätskosten und Budgeteinschränkungen berücksichtigen [8]. Alternative Zugänge werden daher vorgeschlagen, auch direkt von WHO-Forschenden [9].

Eine datengetriebene Annäherung an die Frage, wie viel ein QALY wert ist, kann über den Zusammenhang zwischen Lebenserwartung und Gesundheitsausgaben pro Kopf gemacht werden [10]. Wenngleich der kausale Zusammenhang zwischen diesen Größen nicht klar ist, zeigt sich in vielen Ländern, dass steigende Gesundheitsausgaben mit einer längeren Lebenserwartung einhergehen. Diese Steigung kann als „Zahlungsbereitschaft“ interpretiert werden, also als das Geld, das ein Land im Durchschnitt für die Verlängerung der Lebenserwartung um ein Jahr ausgibt. Für Länder mit hohem Einkommen wie das Vereinigte Königreich oder Deutschland ergeben sich dabei Werte von etwa 50.000 US-Dollar (Basisjahr 2019). In 168 von 174 untersuchten Ländern ergab sich dann ein Schwellenwert von weniger als dem 1‑Fachen des BIP p.K.

Um typische Werte für ICERs zu verstehen, betrachten wir eines der am häufigsten untersuchten Beispiele in der Literatur: die Kosteneffektivität von Brustkrebsscreenings [11]. Diese Intervention wurde in vielen Ländern und Zeiträumen untersucht. Klarerweise hängt die Kosteneffektivität einer neuen Screeningstrategie von der bereits implementierten Strategie ab. Unterschiede in Altersgruppen und Screeningintervallen erschweren die Vergleichbarkeit der Resultate. Ein systematisches Review zeigte jedoch, dass die meisten Studien zu vergleichbaren Ergebnissen kommen: Die Kosten pro gewonnenes Lebensjahr liegen meist zwischen 5000 € und 10.000 €. Es ist jedoch nicht klar, ob diese Vergleichbarkeit der Kosten auch für Studien zu Impfprogrammen für saisonale Atemwegserkrankungen gilt.

In diesem Review stellen wir aktuelle Arbeiten zur Kosteneffektivität von Impfprogrammen zur Prävention der „großen Drei“ der saisonalen Atemwegserkrankungen – Influenza, SARS-CoV‑2, RSV – vor und diskutieren methodische Ansätze sowie deren Stärken und Schwächen.

Die Bewertung von ICERs erfordert eine enorme Datenmenge. Es sind nicht nur umfassende Daten zur klinischen Wirksamkeit und zu den Nebenwirkungen der präventiven Impfungen erforderlich, sondern auch detaillierte Informationen über mögliche Folgeerkrankungen und die damit verbundenen Kosten. Bei Impfungen kommt eine weitere spezifische Herausforderung hinzu: Neben der direkten Schutzwirkung können Impfungen auch eine indirekte Wirkung haben, indem sie die Wahrscheinlichkeit einer Virusübertragung verringern. Dieser indirekte Effekt skaliert jedoch nicht linear mit der Anzahl der verabreichten Impfungen, sondern hängt von der Impfquote und dem bereits bestehenden Immunstatus der Bevölkerung ab.

Für eine präzise Kosteneffektivitätsanalyse von Impfungen müssen daher epidemiologische Modelle eingesetzt werden, die auch den Nutzen durch indirekte Schutzwirkungen berechnen. Dies erhöht sowohl den Datenbedarf als auch die Komplexität der Annahmen, die für verlässliche Schätzungen erforderlich sind. Dies ist besonders relevant für die SARS-CoV-2-Impfung, bei der die indirekten Schutzwirkungen über die Zeit mitunter deutlich abnehmen [12]. Sollten diese indirekten Effekte nicht mitberücksichtigt werden, ist davon auszugehen, dass die Ergebnisse die tatsächliche Kosteneffektivität unterschätzen und in diesem Sinne als konservative Szenarien betrachtet werden können.

Die Literaturrecherche wurde über PubMed durchgeführt. Um gezielt Arbeiten zu identifizieren, die eine Kosteneffektivitätsanalyse von Impfungen, inklusive indirekter Schutzwirkungen, ermöglichen, wurde eine Suchstrategie für Arbeiten mit epidemiologischen Modellen gewählt.^1^ Weiters wurden die Referenzen dieser Arbeit berücksichtigt und noch zusätzlich Studien nach Relevanz ausgewählt, die andere Modellierungsansätze verwenden. Um einen genaueren Einblick in das Zustandekommen der Resultate zu ermöglichen, wird für jede Erkrankung exemplarisch eine Studie detaillierter vorgestellt.

## Influenza

Die PubMed-Suche identifizierte 23 Studien seit 2013, welche die Suchkriterien erfüllten, 16 davon waren keine Reviews und führten umfangreiche Modellierungen zur Kosteneffektivität verschiedener Influenza-Impfstrategien in unterschiedlichen Ländern und Bevölkerungsgruppen durch. Ein zentrales Thema ist der Vergleich zwischen quadrivalenten (QIV) und trivalenten Influenza-Impfstoffen (TIV). Beispielsweise ermittelten Zeevat et al. in den Niederlanden, dass die Einführung von QIV gegenüber TIV zu einem inkrementellen Kosten-Effektivitäts-Verhältnis (ICER) von 20.000 € pro gewonnenem QALY führt [13]. Konsistent damit, berechneten Brogan et al. für die USA einen ICER von 28.000 USD/QALY zugunsten von QIV im Vergleich zu TIV [14]; ähnliche Ergebnisse liegen für brasilianische Kinder im Alter von 6 Monaten bis 5 Jahren [15] und für Kanada [16] vor.

Ein weiterer Schwerpunkt liegt auf der Impfung spezifischer Altersgruppen. De Boer et al. bewerteten die pädiatrische Influenza-Impfung in den Niederlanden mit einem ICER von 15.000 € pro QALY als kosteneffektiv [17]. Shin et al. untersuchten die Ausweitung des nationalen Influenza-Impfprogramms in Südkorea auf ältere Erwachsene und fanden einen ICER von 10.000 USD/QALY, was auf positive gesundheitliche Auswirkungen für die gesamte Bevölkerung hindeutet [18].

Die Einführung neuer Impfstofftechnologien wird ebenfalls thematisiert. Kohli et al. analysierten die Kosteneffizienz eines zellbasierten Influenza-Impfstoffs für Erwachsene im Vereinigten Königreich und fanden einen ICER von 25.000 britischen Pfund pro QALY [19]. Eine weitere Studie des Autorenteams kam zu dem Schluss, dass ein adjuvantierter QIV kostensparend im Vergleich zu einem hochdosierten QIV ist unter der Annahme ähnlicher Wirksamkeit [20]. Zu einem vergleichbaren Schluss kamen Kohli et al. beim Vergleich eines MF59-adjuvantierten mit anderen QIVs bei älteren Erwachsenen in Deutschland [21]. Nguyen et al. nutzten ein dynamisches Modell, um die epidemiologischen und ökonomischen Auswirkungen eines zellbasierten quadrivalenten Influenza-Impfstoffs in den USA zu bewerten und ermittelten einen ICER von 30.000 USD/QALY [22].

Regionale Unterschiede in der Impfstoffwahl und deren wirtschaftliche Auswirkungen werden ebenfalls untersucht. DePasse et al. verwendeten agentenbasierte Modelle, um die Krankheitslast und Kosteneffizienz verschiedener Impfstofftypen in den USA zu vergleichen, und stellten fest, dass die Wahl des Impfstoffs die Krankheitslast und Kosteneffizienz beeinflussen kann, mit ICER-Werten zwischen 20.000 USD/QALY und 50.000 USD/QALY, abhängig von der Region und dem gewählten Impfstoff [23, 24].

Weitere Studien unterstreichen die Bedeutung der Auswahl des geeigneten Influenza-Impfstoffs und der Zielgruppen für Impfprogramme, um sowohl gesundheitliche als auch ökonomische Vorteile zu maximieren. Die Ergebnisse variieren je nach regionalen Gegebenheiten [25], Altersgruppen [26, 27] und verfügbaren Impfstofftypen [28], was die Notwendigkeit einer sorgfältigen Planung und Bewertung von Impfstrategien hervorhebt.

Um einen umfassenderen Einblick in die Durchführung solcher und ähnlicher Kosteneffektivitätsanalysen der jährlichen Influenza-Impfung zu geben, betrachten wir nun die Arbeit von De Luca et al. aus dem Jahr 2023 [29] im Detail. Untersucht wurde die Kosteneffektivität der Influenza-Impfung in den USA zwischen 2010 und 2019. Grundlage war eine universelle Impfempfehlung für alle Personen ab einem Alter von 6 Monaten.

Zur Bewertung wurde ein epidemiologisches Zustandsmodell verwendet, bei dem ungeimpfte Patient*innen 2 unterschiedliche Verläufe nehmen können. Im ersten Szenario (keine Impfung) besteht eine gewisse Wahrscheinlichkeit, dass keine Influenza-Erkrankung auftritt. Wenn jedoch eine Erkrankung eintritt, werden verschiedene Schweregrade definiert, von unkomplizierten Verläufen über die Inanspruchnahme ambulanter Gesundheitsleistungen bis hin zu einer Influenza-assoziierten Hospitalisierung oder einem Influenza-assoziierten Todesfall.

Im Falle einer Impfung wird überprüft, ob Nebenwirkungen auftreten, und wenn ja, welcher Art diese sind – von lokalen Reaktionen an der Einstichstelle über systemische Reaktionen, Anaphylaxie bis hin zum Guillain-Barré-Syndrom. Nach dem Auftreten dieser Nebenwirkungen durchlaufen die Patient*innen das gleiche Zustandsmodell wie im ungeimpften Fall, allerdings mit veränderten Wahrscheinlichkeiten für das Auftreten von Komplikationen und deren Schweregrade.

Für jeden dieser Zustände werden Wahrscheinlichkeiten aus der relevanten Literatur übernommen, wobei viele davon altersabhängig sind. Es müssen nicht nur die Eintrittswahrscheinlichkeiten dieser Ereignisse quantifiziert werden, sondern auch die damit verbundenen Kosten. Das Modell umfasst mehr als 50 Parameter, die alle Unsicherheiten aufweisen.

Die Ergebnisse der Analyse zeigen, dass die Kosteneffektivität mit dem Alter und Risiko variiert. Für Personen über 50 Jahren mit erhöhtem Risiko (z. B. Asthma, Diabetes oder Herzerkrankungen) ist die Impfung sogar kostensparend. Das bedeutet, dass die Impfkosten in dieser Altersgruppe durch die Einsparungen, die durch die Vermeidung schwerer Krankheitsverläufe entstehen, mehr als ausgeglichen werden.

Für Nichtrisikogruppen im Alter zwischen 18 und 49 Jahren lagen die Kosten pro QALY im Basisszenario bei etwa 200.000 USD und damit über den meisten Schwellen der Zahlungsbereitschaft. Für alle anderen Gruppen liegt die jährliche Routineimpfung jedoch unter den typischen Schwellenwerten.

Der Einfluss der Unsicherheit der Parameter wird häufig durch sogenannte Tornado-Diagramme untersucht. Dabei werden die einzelnen Parameter innerhalb ihrer Schwankungsbreiten variiert und die entsprechende Varianz in den Ergebnissen (z. B. Kosten pro QALY) gemessen. Diese Unsicherheitsbereiche werden der Größe nach sortiert dargestellt, was oft eine Trichter- oder Tornado-Form ergibt. Auf diese Weise können die Parameter, die am stärksten zur Unsicherheit beitragen, leicht identifiziert werden.

In der Arbeit von De Luca et al. zeigte sich die größte Unsicherheit in der Wahrscheinlichkeit einer Influenza-Infektion. Diese wurde über verschiedene Größenordnungen hinweg in den unterschiedlichen Altersgruppen variiert, entsprechend den Erfahrungen mit unterschiedlich starken Infektionswellen. In Risikogruppen überstieg die ICER einen Wert von 100.000 USD pro QALY am oberen Ende der Schwankungsbreite, was schwache Influenza-Saisons widerspiegelt. Bei Personen über 65 Jahren blieb die Impfung jedoch auch bei einer Impfstoffeffizienz von nur 4 % unter diesem Grenzwert.

Als Limitation ist anzumerken, dass De Luca et al. keine indirekte Schutzwirkung berücksichtigten. Je höher die Durchimpfungsrate, desto stärker der Effekt der Bevölkerungsimmunität und damit die zusätzliche Vermeidung von Influenza-Infektionen. Die Ergebnisse sind daher als konservative „Worst-Case“-Schätzungen zu betrachten.

Die Bedeutung indirekter Schutzwirkungen wurde auch in einem aktuellen systematischen Review betont, das alle Studien zur Kosteneffektivität von Influenza-Impfungen bei Kindern in Europa untersuchte [30]. Es zeigte sich in sämtlichen Untersuchungen eine positive Kosteneffektivität, wobei ein wesentlicher Teil dieser Kosteneffizienz auf die indirekte Schutzwirkung zurückzuführen war.

In Deutschland ergab eine Studie zur Kosteneffektivität von Impfungen bei Kindern im Alter von 2–9 Jahren mit einem quadrivalenten Grippeimpfstoff und einer angenommenen Impfrate von 40 % eine ICER von 998 € pro QALY im Vergleich zu einer Strategie, nach der nur Personen über 60 Jahren geimpft werden [31].

## SARS-CoV-2

Zu wenigen anderen Impfungen wurden in so kurzer Zeit so viele Kosteneffektivitätsanalysen durchgeführt wie bei der SARS-CoV-2-Impfung. Aufgrund der Dynamik der Pandemie ist es jedoch entscheidend, bei diesen Analysen einzelne Pandemiephasen zu betrachten.

Zum Zeitpunkt des Roll-outs der Impfung traf das Virus auf eine immunologisch naive Bevölkerung, was erhebliche Auswirkungen auf die Kosteneffizienz hatte. Es überrascht daher nicht, dass ein systematisches Review von 25 Studien zum Roll-out zu dem Schluss kam, dass die SARS-CoV-2-Impfung in allen Analysen entweder kosteneffektiv oder kostensparend war [32]. Diese Untersuchungen sind jedoch nicht mehr aussagekräftig, wenn es um die Bewertung der aktuellen Situation und SARS-CoV-2-Impfempfehlungen geht.

Kosteneffektivitätsanalysen zur SARS-CoV-2-Impfung in der heutigen epidemiologischen Lage, in der ein großer Teil der Bevölkerung bereits eine Basisimmunität besitzt und die zirkulierenden Varianten seltener zu schweren Verläufen führen, sind in der Literatur wesentlich seltener. Unsere Suchstrategie identifizierte 23 Arbeiten. Von diesen behandelten jedoch nur 3 Studien die Kosteneffektivität von aktualisierten Impfstoffen und weitere 3 die Kosteneffektivität der Booster-Impfungen [33–35].

Wir fokussieren hier auf die Arbeiten, welche die für Herbst 2023 oder 2024 aktualisierten COVID-19-mRNA-Impfstoffe behandeln. Eine Studie von Joshi et al. verwendet ein Susceptible-Exposed-Infected-Recovered-(SEIR-)Modell, um für Deutschland zu schätzen, dass eine Impfkampagne mit dem Moderna-Impfstoff mRNA-1273.815 im Vergleich zu keiner zusätzlichen Impfung etwa 1.697.900 symptomatische Infektionen, 85.400 Krankenhausaufenthalte und 4100 Todesfälle verhindern könnte [36]. Im Vergleich zum Pfizer-BioNTech-Impfstoff XBB.1.5 BNT162b2 könnten zusätzlich 90.100 symptomatische Infektionen, 3500 Krankenhausaufenthalte und 160 Todesfälle vermieden werden.

Eine umfassende Studie dieser Art untersuchte die ICERs der Pfizer-BioNTech- und Moderna-Impfungen im Herbst 2023 in den USA und verglich diese mit einem Szenario ohne Auffrischungsimpfung [37].

Kohli et al. verwendeten ein epidemiologisches SEIRS-Modell, das die Bevölkerung in suszeptible, exponierte, infizierte und genesene Personen unterteilt. Genesene verlieren ihre Immunität im Laufe der Zeit und werden erneut suszeptibel. Das Modell wurde mit Daten bis zum Sommer 2023 kalibriert. Analysiert wurden symptomatische Infektionen, Covid-19-assoziierte Hospitalisierungen und Todesfälle, wobei die entsprechenden Kosten und verlorenen QALYs mithilfe von Entscheidungsbäumen modelliert wurden.

Das Modell berücksichtigt zahlreiche Immunisierungspfade: Personen können ungeimpft, einmal geimpft, 2‑ oder 3‑mal geboostert sein oder bereits eine Auffrischungsimpfung vor Herbst 2023 erhalten haben. Für jeden dieser Zustände gibt es ein eigenes Kompartment suszeptibler Personen, aus denen sich jeweils exponierte, infizierte und genesene Kompartments mit unterschiedlichen Übergangsraten ableiten lassen. Insgesamt ergeben sich 24 Kompartments, noch bevor Altersabhängigkeiten berücksichtigt werden.

Zur Bewertung der Vakzineffektivität wurden unterschiedliche Szenarien betrachtet, die auch die Unsicherheit hinsichtlich neuer Varianten berücksichtigen. Bei hoher Vakzineffektivität, die bei antigenisch ähnlichen Varianten angenommen wird, geht man von einer anfänglichen Schutzwirkung von über 80 % aus. In Szenarien mit unterschiedlicheren Varianten sinkt die Effektivität auf unter 60 % bzw. im Worst-Case-Szenario auf etwa 30 %.

Auf Grundlage dieser anfänglichen Effektivität wurden unterschiedliche Abnahmeraten modelliert. Selbst im optimistischsten Szenario wird davon ausgegangen, dass die Schutzwirkung gegen Infektionen nach einem Jahr auf knapp über 30 % sinkt. Im pessimistischsten Szenario gibt es nach April 2024 praktisch keine Schutzwirkung mehr.

Die Schutzwirkung vor schweren Verläufen wurde ebenfalls modelliert, wobei von anfänglich höheren Werten (80–90 %) ausgegangen wird, die langsamer abnehmen als der Schutz vor Infektionen. Selbst im pessimistischsten Szenario beträgt die Schutzwirkung nach einem Jahr noch etwa 70 %.

Die klinischen Folgen wurden mithilfe detaillierter Entscheidungsbäume modelliert, die nach Alter und Immunisierungsstatus variieren. Für alle symptomatischen Infektionen wurde das Risiko einer Myokarditis berücksichtigt. Bei hospitalisierten Patient*innen wurde zwischen Aufnahme auf der Normalstation, der Intensivstation und der Intensivstation mit Beatmung unterschieden, wobei für jede dieser Gruppen unterschiedliche Sterbewahrscheinlichkeiten im Krankenhaus oder kurz nach Entlassung modelliert wurden. Zusätzlich wurde das Risiko von Long Covid bei allen Infizierten berücksichtigt.

Diese Analysen sind extrem datenintensiv. Besonders die Auswahl der modellierten klinischen Folgen ist entscheidend, um die relevantesten kostentreibenden Ereignisse abzubilden. Ein Fokus lag auf SARS-CoV-2-assoziierter Myokarditis, deren Kosten auf 14.000 USD pro Ereignis geschätzt werden. Die Kosten für stationäre Behandlungen reichen von etwa 15.000 USD für einen Aufenthalt auf der Normalstation über 27.000 USD bei einem Intensivaufenthalt bis zu 70.000 USD bei Beatmung.

Die Kosten für postakute Behandlungen wurden über einen Zeitraum von 180 Tagen für alle Patient*innen modelliert. Diese Kosten wurden basierend auf dem Alter und der Schwere der vorangegangenen Infektion geschätzt, wobei für Long Covid eine pauschale Kostenannahme gemacht wurde. Diese variieren zwischen 70 USD für nichthospitalisierte Kinder im Alter von 0–4 Jahren und 700 USD für hospitalisierte Personen im Alter von 50–64 Jahren.

Die Schätzungen zu den verlorenen QALYs pro Infektion variieren ebenfalls stark zwischen den verschiedenen Gruppen. Während symptomatische Infektionen ohne Krankenhausaufenthalt mit einem Verlust von 0,003 QALYs zu Buche schlagen, gehen pro Fall einer postakuten Hospitalisierung 0,122 QALYs verloren.

Hospitalisierungsraten, Wiederaufnahmeraten sowie Sterblichkeitsraten im Krankenhaus und nach Entlassung wurden ebenfalls nach Altersgruppe und Schwere des Verlaufs geschätzt. Die Sterblichkeitsrate steigt mit dem Alter stark an: Bei über 85-Jährigen beträgt die Sterblichkeit auf der Normalstation etwa 20 %, auf der Intensivstation 30 % und bei Beatmung 70 %. In der Altersgruppe der 50- bis 64-Jährigen betragen diese Werte 1 %, 4 % bzw. 46 %. Die Betrachtung der QALYs ermöglicht es, die stark altersabhängige Anzahl der verlorenen Lebensjahre zu berücksichtigen.

Verlorene QALYs durch Impfnebenwirkungen wurden ebenfalls modelliert. Für Anaphylaxie und impfstoffinduzierte Myokarditis/Perikarditis wurden jeweils 0,0019 verlorene QALYs geschätzt, bei einer Inzidenz von 0,0005 % bzw. 0,0018 %, was Kosten von etwa 3500 USD pro Ereignis verursacht. Häufiger treten lokale und systemische Nebenwirkungen vom Grad 3 auf, die mit einer Wahrscheinlichkeit von 4,9 % bzw. 7,6 % auftreten und dabei 0,0011 bzw. 0,0003 QALYs kosten und etwa 10 USD verursachen.

Die Ergebnisse der epidemiologischen Modellierung zeigen erwartungsgemäß, dass die Zahl der symptomatischen Infektionen im Szenario ohne Auffrischungsimpfung deutlich höher ist als im Szenario mit Impfung. Die Hospitalisierungsrate pro 100.000 Personen sinkt im Impfszenario in der Altersgruppe über 65 Jahre von 1000 auf 600 und in der Altersgruppe von 50 bis 64 Jahren von etwa 400 auf 300. Die Zahl der Todesfälle pro 100.000 sinkt von 180 auf 100 bei den über 65-Jährigen und von 50 auf 40 bei den 50- bis 64-Jährigen.

Im Basisszenario ergab sich für das Moderna-Vakzin eine ICER von 7700 USD pro QALY. Betrachtet man nur Personen über 65 Jahre, so ergibt sich eine ICER von 1800 USD pro QALY. Im direkten Vergleich zwischen dem Moderna- und dem Pfizer-BioNTech-Impfstoff wurde der Pfizer-Impfstoff mit geringfügig höheren Kosten und etwas mehr verlorenen QALYs bewertet. Bemerkenswert ist, dass 94 % der verlorenen QALYs auf postakute Folgen zurückzuführen waren.

In der Sensitivitätsanalyse wurden die größten Unsicherheiten hinsichtlich der Vakzineffektivität gegen Infektion und der Möglichkeit des Entstehens einer neuen immunflüchtigen Variante festgestellt. Selbst in den pessimistischsten Annahmen lag die ICER jedoch immer unter 40.000 USD.

Das weitgehend gleiche Autorenteam untersuchte auch die Kosteneffizienz einer Auffrischungsimpfung im Herbst 2024 im Vereinigten Königreich [38]. Das methodische Vorgehen war ähnlich und die Studie ist derzeit als Preprint verfügbar, jedoch noch nicht „peer-reviewed“. Die Modellierung legt nahe, dass eine Impfkampagne symptomatische Infektionen um 19 %, Hospitalisierungen um 38 %, Long-Covid-Fälle um 19 % und Todesfälle um 43 % reduzieren kann. Die ICER für eine Impfkampagne bei Personen über 65 Jahren wurde mit etwa 10.000 € pro QALY berechnet, bei über 50-Jährigen mit etwa 12.000 € pro QALY.

Zusammenfassend lässt sich sagen, dass die SARS-CoV-2-Impfung in den jeweiligen Altersgruppen nach wie vor kosteneffektiv ist, trotz der inzwischen höheren Immunisierung in der Bevölkerung. Die Impfung reduziert weiterhin signifikant schwere Krankheitsverläufe, postakute Folgen und Todesfälle auf kosteneffektive Weise.

## RSV

Im Jahr 2023 wurden 2 RSV-Impfstoffe von den Firmen GSK und Pfizer zugelassen. Erste Schätzungen zur Kosteneffizienz dieser Impfstoffe liegen bereits vor. Die Literatursuche ergab 5 Studien für RSV, von denen jedoch keine diese neuen Impfstoffe behandelt. Eine Studie von Hutton et al. modellierte die Auswirkungen einer Impfung auf die Krankheitslast über einen Zeitraum von 2 Jahren [39]. Die Kosten pro gewonnenes qualitätsadjustiertes Lebensjahr (QALY) variieren dabei je nach Impfstoff und Altersgruppe. Für Erwachsene über 65 Jahren belaufen sich die Kosten pro QALY auf etwa 160.000 USD für den GSK-Impfstoff bzw. 150.000 USD für den Pfizer-Impfstoff. Bei Personen im Alter von 60 bis 65 Jahren sind die Kosten jedoch mehr als doppelt so hoch, was auf eine geringere Krankheitslast in dieser Altersgruppe zurückzuführen ist.

Das Modell zeigt, dass die Kosteneffektivität stark von der Impfstoffeffizienz, den Impfstoffkosten und der Krankheitsinzidenz abhängt. Insbesondere niedrigere Impfstoffkosten und eine anhaltende Schutzwirkung über mehr als 2 Jahre würden die Kosten-Nutzen-Relation deutlich verbessern. Sensitivitätsanalysen ergaben außerdem, dass eine höhere Krankheitsinzidenz die Kosteneffizienz erheblich steigern könnte.

Ob die RSV-Impfung als kosteneffektiv betrachtet werden kann, hängt von der Bereitschaft ab, für ein gewonnenes QALY zu zahlen. Schätzungen zufolge müssten die Kosten pro Impfdosis unter 200 USD liegen, damit die ICER für Personen über 65 Jahren auf unter 100.000 USD pro QALY sinkt. Für eine Impfstrategie, die alle Personen ab 60 Jahren umfasst, müssten die Kosten zwischen 150 USD und 200 USD pro Dosis liegen. Zudem wird angenommen, dass die RSV-Impfung in der Altersgruppe über 75 Jahren bereits heute unter dem Schwellenwert von 100.000 USD pro QALY liegt.

## Schlussfolgerungen

Gesundheitsökonomische Analysen bestätigen die Kosteneffizienz der jährlichen Influenza-Impfung sowie der SARS-CoV-2-Auffrischungsimpfungen in den Jahren 2023 und 2024. Besonders in Hochrisikogruppen erweisen sich die Impfungen teilweise als kostensparend. Bei der RSV-Impfung ist die Lage weniger eindeutig. Hier hängt es stark von der Zahlungsbereitschaft und der Altersgruppe ab, ob die Impfung als kosteneffektiv bewertet werden kann.

Es muss jedoch betont werden, dass diese Studien auf Annahmen zur Bevölkerungsstruktur (z. B. Altersstruktur, Sozioökonomie) und den jeweiligen Gesundheitssystemen (z. B. Behandlungskosten) basieren. Daher ist unklar, inwieweit die Ergebnisse zwischen den verschiedenen Ländern vergleichbar sind, insbesondere in Bezug auf Kosten und Ressourcenverbräuche. Da die meisten der betrachteten Länder aus Europa und Nordamerika stammen, ist nicht zu erwarten, dass sich die Resultate so stark unterscheiden, wie es etwa bei einem Vergleich zwischen Ländern mit hohem und niedrigem Einkommen der Fall wäre. Dennoch wäre es wünschenswert, solche Analysen auch umfassender für Länder des deutschsprachigen Raums durchzuführen.

Schätzungen berücksichtigen zahlreiche Informationen zu Kosten (direkte medizinische Kosten, Folgekosten, Produktivitätsverluste usw.) und Nutzen (kurzfristig, langfristig, direkt, indirekt). Dies unterstreicht die Notwendigkeit systematischer Reviews und Sensitivitätsanalysen. Ebenso wäre es von Vorteil, Daten aus verschiedenen Ländern miteinander vergleichen zu können.

Diese Schätzungen sind jedoch auch äußerst datenintensiv. Für die Kalibrierung der verwendeten Modelle müssen umfangreiche Gesundheitsdaten der Bevölkerung verknüpft werden, einschließlich Informationen zu Infektionskrankheiten, möglichen Folgeerkrankungen Monate nach der Infektion, Impfdaten und Sterbedaten. In vielen Ländern steht dem jedoch eine nach wie vor stark fragmentierte Gesundheitsdatenlandschaft gegenüber, die solche Verknüpfungen erschwert oder unmöglich macht. Dies führt dazu, dass man sich in gesundheitsökonomischen Analysen oft im „Blindflug“ befindet und darauf hoffen muss, dass Ergebnisse aus Ländern mit besserer Dateninfrastruktur übertragbar sind.

Initiativen wie der europäische Gesundheitsdatenraum sollten daher mit Nachdruck weiter vorangetrieben werden, um eine Kultur der evidenzbasierten Entscheidungsfindung zu etablieren und die Integration von Gesundheitsdaten zu verbessern.

## Footnotes

1 Gesucht wurde nach „cost-effectiveness“ und „vaccine“ sowie „epidemiological model“ oder „compartmental“ in Titel oder Abstract sowie der entsprechenden Erkrankung.
